# The International Medical Congress

**Published:** 1886-10

**Authors:** 


### THE INTERNATIONAL MEDICAL CONGRESS.

The attitude of a portion of the most eminent men in the medical profession of the United States towards the present organization of the ninth International Congress is the result of circumstances which cannot be properly comprehended by those abroad, who are unacquainted with the influences which brought about their withdrawal from the positions of honor and responsibility to which they were nominated. It is now too late to discuss the merits of their dissatisfaction, but considerations of self-respect still induce most of them to stand aloof from all official connection with the Medical Congress, while some will co-operate in the scientific work of that body. No good end can be subserved by keeping up this issue, and thus presenting to the outside medical world a dissension in the constituents of the American profession that can serve no other purpose than to lower their standing abroad; so that it behooves all who consider themselves as precluded from participation in the proceedings of the International Medical Congress to maintain a position of dignified inactivity and afford no let or hindrance to the progress of the plans now in operation for making it a success.

All members of the profession in this country are alike interested in our recognition abroad, and it behooves them to do nothing which may militate against the status of American medicine. As in our political campaigns, the greatest vigilance and activity is employed by the opposing parties in sustaining their respective platforms as represented by different candidates up to the time of election, when the decision must be regarded as the choice of the whole, so now the opposing views should be reconciled in supporting the measures adopted by the existing organization of the Congress, or at least in not opposing what is being done to secure a satisfactory result. While many have not approved of the course taken by those in charge of this undertaking, yet, it being understood now that the ninth International Congress will be held at the appointed time in the city of Washington, every member of the profession should feel committed to uphold the honor of his brotherhood by promoting the scientific outgrowth from an interchange of sentiments with the representatives of other countries in this reunion of the medical world. To put forth impressions unfavorable to the progress of the work at this stage of affairs is like a bird fouling its own nest, and if there are some who cannot consistently aid in the undertaking, they should at least refrain from doing anything prejudicial to its success.

